# Dynamics of leaching of POPs and additives from plastic in a Procellariiform gastric model: Diet- and polymer-dependent effects and implications for long-term exposure

**DOI:** 10.1371/journal.pone.0299860

**Published:** 2024-03-27

**Authors:** Liesbeth Van Hassel, Georges Scholl, Gauthier Eppe, Claude Poleunisc, Christine Dupont-Gillain, Myra Finkelstein, Cathy Debier

**Affiliations:** 1 Louvain Institute of Biomolecular Science and Technology, Université Catholique de Louvain, Louvain-la-Neuve, Belgium; 2 Mass Spectrometry Laboratory, MolSys Research Unity, Université de Liège, Liège, Belgium; 3 Institute of Condensed Matter and Nanosciences, Université Catholique de Louvain, Louvain-la-Neuve, Belgium; 4 Microbiology & Environmental Toxicology Department, University of California Santa Cruz, Santa Cruz, CA, United States of America; VIT University, INDIA

## Abstract

Procellariiform seabirds are known to have high rates of plastic ingestion. We investigated the bioaccessibility of plastic-associated chemicals [plastic additives and sorbed persistent organic pollutants (POPs)] leached from plastic over time using an *in vitro* Procellariiform gastric model. High-density polyethylene (HDPE) and polyvinyl chloride (PVC), commonly ingested by Procellariiform seabirds, were manufactured with one additive [decabrominated diphenyl ether (PBDE-209) or bisphenol S (BPS)]. HDPE and PVC added with PBDE-209 were additionally incubated in salt water with 2,4,4’-trichloro-1,1’-biphenyl (PCB-28) and 2,2’,3,4,4’,5’-hexachlorobiphenyl (PCB-138) to simulate sorption of POPs on plastic in the marine environment. Our results indicate that the type of plastic (nature of polymer and additive), presence of food (i.e., lipids and proteins) and gastric secretions (i.e., pepsin) influence the leaching of chemicals in a seabird. In addition, 100% of the sorbed POPs were leached from the plastic within 100 hours, while only 2–5% of the additives were leached from the matrix within 100 hours, suggesting that the remaining 95% of the additives could continue to be leached. Overall, our study illustrates how plastic type, diet and plastic retention time can influence a Procellariform’s exposure risk to plastic-associated chemicals.

## Introduction

Plastic ingestion is widespread among seabirds with 80% of studied species affected [1]. Procellariiforms (petrels, albatrosses and shearwaters) rarely regurgitate indigestible items [2] and their digestive morphology prevents ingested plastics larger than 2–5 mm [3] to pass through their digestive tract [4], which results in a larger proportion of species documented with plastic trapped in their stomach compared to other taxa [1]. In addition, Procellariforms have one of the highest documented incidence rates of plastic ingestion, ranging from 50 to 100% depending on the species [1].

Plastic ingestion can harm seabirds, including Procellariforms, by mechanical damage and obstruction [5–7] but also by exposure to plastic-associated chemicals [8]. Broadly, plastic-associated chemicals can be categorized into two groups (i) the plastic matrix, including additives and (ii) persistent organic pollutants (POPs) that sorb to the surface of plastic. Additives are chemicals mixed with polymers during the manufacture of plastic to modify their properties, performances and long-term use (e.g. flame retardants, plasticizers, antioxidants, UV stabilizers, lubricants, dyes and fillers) [9]. Most additives are not covalently bound to the polymeric structure of plastic and leach readily into the surrounding environment [10]. POPs can sorb onto plastic in aquatic environments; plastic has been shown to concentrate POPs, such as polychlorinated biphenyls (PCBs) [11], by factors up to 10^7^ compared to seawater [12]. While numerous studies have documented the prevalence of plastic ingestion in seabirds [1, 13], the exposure risk to plastic-associated chemicals remains poorly understood [14].

One potential risk of exposure to plastic-associated chemicals is endocrine disruption [15–17], which is of concern to affect a seabird’s hormonal and reproductive system [18, 19]. A key data gap in understanding exposure risk is the bioaccessibility of plastic-associated chemicals in a seabird’s digestive tract over time [14]. The few studies that have investigated the leaching of chemicals from plastics ingested by seabirds have only examined the effect of isolated gastric solutions on the leaching of additives (i.e. oil [20–22] or acidic pepsin solution [23, 24]) and the leaching of POPs from plastic has only been studied under gut fluid conditions (i.e. bile solutions [25, 26]). Notably, these investigations have never considered the co-existence of both additives and sorbed POPs. In addition, even though plastic might be present in a Procellariiform’s stomach (proventriculus and/or gizzard chamber) for weeks to months [27, 28], to our knowledge, the temporal leaching of these plastic-associated chemicals from ingested plastic has not been investigated.

In order to assess exposure risk to plastic-associated chemicals, we developed an *in vitro* Procellariiform gastric model (PGM) and evaluated the influence of gastric fluids (acidity and enzymes) and food (lipids and proteins) on the temporal leaching of plastic-associated chemicals (additives and sorbed POPs). We also investigated how different gastric components (e.g. pH, enzymes, proteins and lipids) affected the leaching of these chemicals. We used two plastic polymers known to be commonly ingested by Procellariiforms: high-density polyethylene (HDPE), characterized by a high permeability and free volume, and polyvinyl chloride (PVC), more compact with less free volume [29]. Each polymer was prepared with one additive: either decabrominated diphenyl ether (PBDE-209) or bisphenol-S (BPS), known for their reproductive toxicity [30–35] and distinct chemical properties. To simulate our PGM, plastic pieces were incubated in a different digestive solution every 20 hours for a total of 100 hours. The influence of food composition on the chemical leaching was assessed with two marine oils (salmon and calanus), commonly found in Procellariform stomach oil [36–38]. Our study highlights the pivotal role of food and gastric secretions in modulating the leaching of plastic-associated chemicals and investigates the exposure risk posed by additives compared to sorbed POPs present on the plastic surface.

## Materials and methods

Detailed information about the provenance of chemicals and materials used in this study can be found in the Supporting Information, S1 Protocol.

### Plastic sample preparation

#### Plastic manufacture

**Fig 1:**
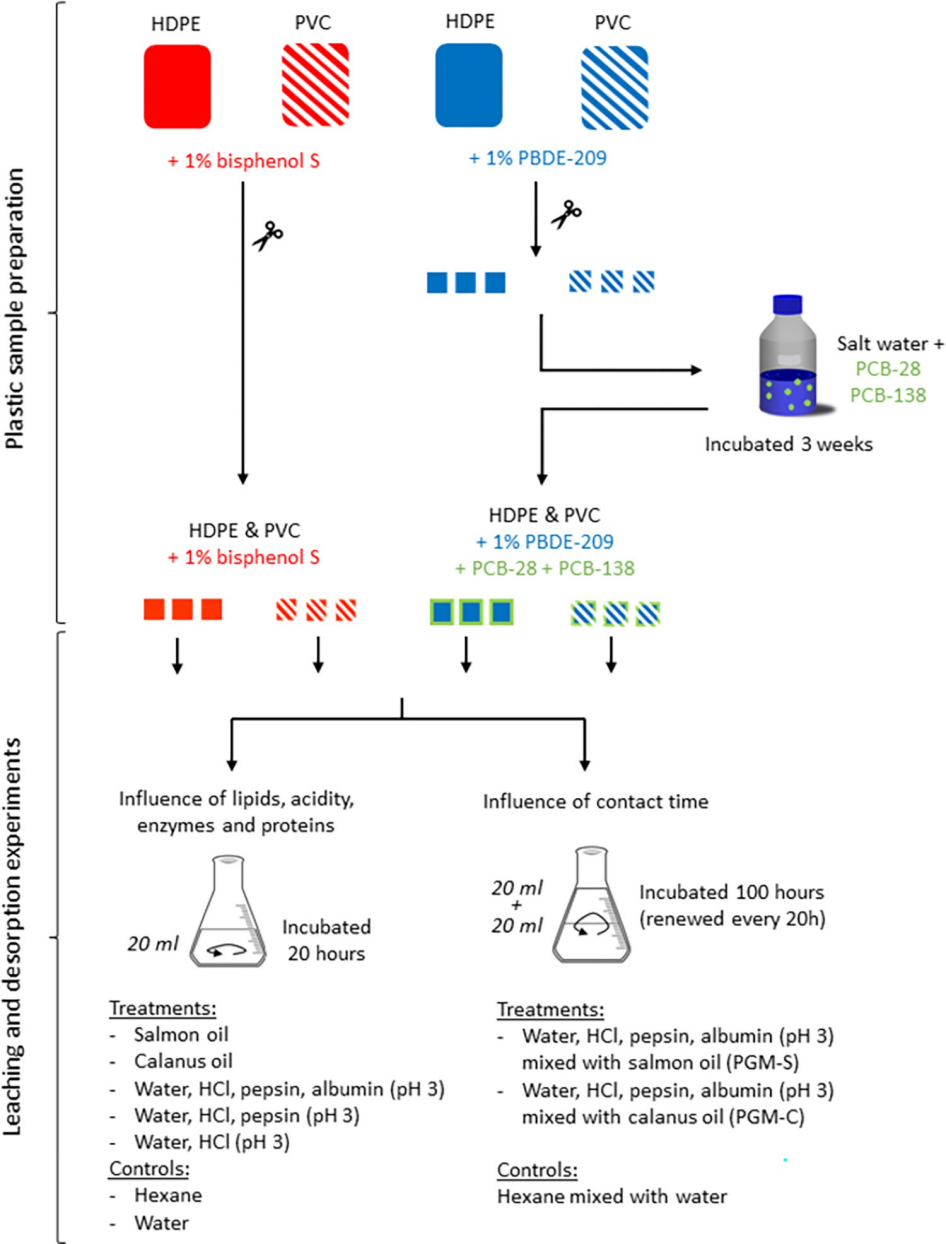
Experimental design. Two HDPE and PVC plastic plates were supplemented with decabrominated diphenyl ether (PBDE-209) or bisphenol S (BPS) at a concentration of 1% (w/w). Plastic plates were cut in 5 mm x 5 mm x 1 mm square pieces. A total of 300 pieces of each polymer with 1% PBDE-209 were incubated in 100 ml of salt water under agitation (150 rpm; RT; in the dark) for three weeks with polychlorinated biphenyl -28 and -138 (PCB-28 and PCB-138). To study the influence of lipids, enzymes, proteins and acidity, ten plastic pieces (HDPE or PVC) containing either 1% BPS or 1% PBDE-209 + PCBs-28 and -138 were exposed to either (i) salmon (ii) calanus oil, (iii) an acidic albumin-pepsin solution (iv) an acidic pepsin solution or (v) acidic water for 20 hours at 38°C. Hexane and milli-Q water were used as controls. To study the influence of contact time on the release of chemicals, 10 plastic pieces containing either 1% BPS or 1% PBDE-209 + PCB-28 and -138 were incubated in the Procellariiform gastric model (PGM), containing an acidic albumin-pepsin solution mixed either with salmon oil or calanus oil for 100 hours. This gastric solution was replaced every 20 hours. Controls of water mixed with hexane were run in parallel.

Four plastic plates (110 mm x 110 mm x 1 mm) were manufactured by Certech R&D Partner in Chemistry (Seneffe, Belgium) (S2 Protocol) (Fig 1). One HDPE and one PVC plate were supplemented with PBDE-209 as an additive at a concentration of 1% (w/w). PBDE-209 is a highly hydrophobic flame retardant (log K_ow_ = 9.97 [39]) which has been detected in ingested plastics and Procellariiform tissues [20, 40, 41]. The two other HDPE and PVC plates were supplemented with BPS as an additive at the same concentration (1%, w/w). BPS is a weakly hydrophobic antioxidant (log K_ow_ = 1.65 [42]) used as an analogous of the famous bisphenol-A (BPA), which has been detected in marine plastics [43]. The concentration was chosen according to the typical concentration of flame retardants and anti-oxidants used in polymers [10, 44]. Plastic plates were cut in 5 mm x 5 mm x 1 mm square pieces (surface area of 70 mm^2^), according to the estimated size of plastic items trapped in the gizzard of Procellariiforms (2–10 mm) [3].

#### Time-of-Flight Secondary Ion Mass Spectrometry (ToF-SIMS) analyses

Time-of-Flight Secondary Ion Mass Spectrometry (ToF-SIMS) analyses were performed on HDPE and PVC pieces supplemented with BPS and PBDE-209 to characterize the distribution of additives within the polymer matrices (S1 Fig). ToF-SIMS analyses were performed using a TOF.SIMS^5^ instrument (IONTOF GmbH, Münster, Germany), equipped with a Bi Nanoprobe-LMIG (liquid metal ion gun) and an Ar-GCIB (gas cluster ion beam) primary ion sources (beams oriented at 45° to the surface normal). Depth profiling experiments were conducted using the dual-beam mode. The 30 keV Bi_5_^+^ pulsed ion beam (cycle time of 200 μs) with a raster of analysis of 200 x 200 μm^2^ was used for spectral acquisition. The measured analysis current was ~0.4 pA. The 10 keV Ar_3000_^+^ with a raster set to 450 x 450 μm^2^ was used for sputtering. The measured sputtering current was 4 nA. Charge compensation was achieved with a 20 eV electron flood gun.

#### Sorption of POPs in salt water

A total of 300 pieces of each polymer with 1% PBDE-209 were incubated in 100 mL of salt water (35 g NaCl/L) under agitation [150 rpm; room temperature (RT); in the dark] for three weeks with polychlorinated biphenyl -28 and -138 (PCB-28, a trichlorinated biphenyl and PCB-138, an hexachlorinated biphenyl), two POPs known to be sorbed on plastic in the ocean [26, 45, 46] (Fig 1). The three-week incubation period was chosen according to Nor et al. (2019), who reported that longer incubation time assures a deeper sorption of the chemicals by the polymer, which can be considered as more environmentally relevant than the shorter equilibrium time (24 – 48h) used in prior studies [26]. Salt water was spiked with 129.4 μg/L PCB-28 and 114.3 μg/L PCB-138 resulting in a maximum amount of 43.1 ng PCB-28 and 38.1 ng PCB-138 per plastic piece to approximate an environmentally relevant sorption of PCBs on plastic surface [12, 45–48]. Accordingly, the concentration of sorbed POPs was about ~10 times smaller than the concentration of additives present within the plastic matrix–which is representative of what happens in the environment [49]. After incubation, plastic pieces were removed with a sieve, briefly rinsed with milli-Q water, dried under a chemical-fume hood overnight, and stored in glass vials in the dark at RT. Sorption experiments with PCBs were not performed on plastics containing BPS as an additive as a three-week soaking in salt water may trigger the leaching of BPS from the plastic bulk (log K_ow_ 1.65 for BPS vs 9.97 for PBDE-209 [39, 42]).

### Leaching experiments

#### Procellariiform gastric model (PGM)

A seabird’s stomach is divided in two chambers, the proventriculus and gizzard. Our Procellariiform gastric model (PGM) was based on the conditions of the gizzard (second chamber) where the majority of the plastic remains trapped and is exposed to both chemical and mechanical digestion, increasing the potential leaching of chemicals [50]. In this study, we considered that plastic pieces retained in the gizzard were exposed to a new gastric solution every 20 hours, based on the average time of gastric digestion in Procellariiforms [51–53]. The Procellariiform gastric solution was simulated with a mixture of 20 mL of marine oil and 20 mL of an aqueous solution made of milli-Q water, pepsin (10 g/L) and albumin (10 g/L) adjusted to a pH of 3 by adding first 37% and then 1 M HCl dropwise [54]. Those conditions were chosen according to the volume of stomach oil and the composition of gastric juice in Procellariiforms [21, 23, 55]. Albumin was chosen to represent the food proteins present in the stomach. The albumin concentration used was derived from the average mass of a prey found in a Procellariiform gizzard [54], along with an estimated associated gastric fluid volume of 120 ml [55, 56], assuming a protein concentration of 16% [53]. Procellariform stomach oil consists mainly of triacylglycerols (TAG) and wax esters (WE) (70–99% total lipids) at ratios varying according to the diet [36, 37, 57]. Accordingly, we investigated the effect of two marine oils: a salmon TAG-rich oil and a calanus WE-rich oil. In order to simulate the mechanical action of the gizzard, the Procellariiform gastric solution was continuously stirred with a glass magnetic stir bar at 800 rpm, at 38°C (the body temperature of Procellariiforms [58]). After incubation, samples were stored in pre-cleaned glass vials at -20°C in the dark prior to analyses.

#### Influence of lipids, acidity, enzymes and proteins on chemical leaching

Gastric solution components were tested individually to investigate the effect of food (lipids and proteins), digestive enzymes (pepsin) and acidity on the leaching of chemicals from plastic. Ten plastic pieces of HDPE (mass = ± 0.23 g) or ten plastic pieces of PVC (mass = ± 0.39 g) containing either 1% BPS or 1% PBDE-209 + PCBs-28 and -138 (sorbed) were exposed to (i) salmon oil, (ii) calanus oil, (iii) an acidic aqueous solution containing both pepsin and proteins [milli-Q water, pepsin (10 g/L) and albumin (10 g/L), pH 3], (iv) an acidic aqueous solution containing only pepsin [milli-Q water with pepsin (10 g/L; pH 3)] or (v) acidic water (pH 3) for 20 hours at 38°C (800 rpm; 10 pieces/20 mL of solution; Fig 1).

#### Influence of contact time with gastric solution

Ten plastic pieces of HDPE (mass = ± 0.23 g) or ten plastic pieces of PVC (mass = ± 0.39 g) containing either 1% BPS or 1% PBDE-209 + PCB-28 and -138 (sorbed) were incubated in the PGM, combining the acidic albumin-pepsin solution and salmon (PGM-S) or calanus (PGM-C) oil for 100 hours [800 rpm; 10 pieces/40mL of solution; 50:50 (v/v); Fig 1] to investigate the leaching and desorption behavior of chemicals over time. The gastric solution was replaced every 20 hours, the average time of gastric digestion in Procellariiforms [51–53]. The solid-to-liquid ratio was based on the average number of plastic pieces and the digestive fluid volume in the stomach of Procellariiforms [55, 59].

#### Quality controls

In order to ensure the reliability and accuracy of our experimental results, rigorous quality control measures were implemented throughout the study. Glassware used in POPs sorption and leaching experiments was pre-washed three times with analytical-grade hexane and analytical-grade methanol. Glassware intended for chemical analysis was baked in a muffle furnace at 450°C for > 4 h prior to use. Additionally, plastic-free control solutions were processed and analyzed in parallel to detect any potential contamination. All solutions contained no detectable chemicals, with the exception of marine oils, which contained a minimal amount of PCBs (< 2.5 μg/g) that was subsequently subtracted from the total concentrations measured. Hexane and water were used as positive controls as they are known to promote the leaching of hydrophobic chemicals (PBDEs and PCBs; hexane) [20, 41, 60] and more water-soluble chemicals (bisphenol analogous) [61].

### Chemical analyses

#### PBDE-209, PCB-28 and– 138

Aqueous samples were liquid-liquid extracted according to the following procedure: 1 mL of n-hexane was added to 1 mL of sample and 10 μL of 200 pg/μL ^13^C-labelled PCB solution (PCB-28, -52, -101, -138, -153, -180) as well as 10 μL of 2,500 pg/μL ^13^C-labelled PBDE solution (PBDE-28, -47, -77, -99, -100, -138, -153, -154, -183, -209) were added for quantification and used for method recovery (range of accepted recoveries were 60–120%). The solution was vortexed for 30 seconds and settled for 5 minutes before recovering the upper n-hexane phase. Two additional liquid-liquid extractions were performed following the same procedure. For the oils, 10 mL n-hexane were added to 100 μL of sample and 10 μL of 200 pg/μL ^13^C-labelled PCB solution as well as 10 μL of 2,500 pg/μL ^13^C-labelled PBDE solution were added as internal standards. The resulting solutions (3 mL of hexane for aqueous samples and 10 mL of hexane for lipidic samples) were purified by solid-liquid adsorption chromatography (S3 Protocol). After evaporation under a gentle dried-air stream to approximately 90 μL, 10 μL of a 200 pg/μL ^13^C-labelled PCB-80 solution was added as internal standard for instrument calibration. Final concentration data were normalized to their recovery standard. Operating parameters regarding the instrumental analysis are detailed in S3 Protocol.

#### BPS

Five μL of bisphenol S-d_8_ at 100 μg/mL water was added to 500 μL of aqueous sample as an internal standard. The solution was vortexed and centrifuged and 100 μL were transferred into a UPLC vial for injection. For hexane and oil samples, 5 μL of 100 μg/mL bisphenol S-d_8_ were added to 1 mL of sample as an internal standard. Five mL of hexane was added to the sample and mixed thoroughly. A volume of 2 mL of methanol was then added to the solution, shaken thoroughly, and centrifuged at 10,000 rpm for 2 minutes. The lower phase was filtered using a syringe filtration (0.2 μm filter) into a UPLC vial for injection. Operating parameters regarding the instrumental analysis are detailed in S3 Protocol.

### Statistical analyses

The percentage of additive leached in each solution was calculated as the ratio of the mean additive concentration measured in the digestive medium (n = 3) and the total concentration of additive added to the polymer (10,000 μg/g). The percentage of PCBs leached in each medium was calculated as the ratio of the mean PCB concentrations (PCB-28 or PCB-138) measured in the digestive medium (n = 3) and the mean PCB concentrations measured in hexane (positive control; n = 3) (S4 Protocol).

Unequal variance bilateral t-tests were conducted to compare the percentage of chemicals leached between HDPE and PVC when the influence of lipids, acidity, enzymes and proteins on the leaching of chemicals was studied. To assess differences in chemical leaching between each treatment, a Welch analysis of variance (ANOVA) was used. Subsequently, individual pairwise comparisons between treatments were performed using t-tests with a Bonferroni correction to account for multiple comparisons. These analyses elucidated how different treatments affected chemical leaching from the plastic materials. Regarding the influence of contact time on the leaching of chemicals, Welch ANOVA was employed to assess the differences in chemical leaching over time between the two treatments (PGM-S and PGM-C) while accounting for potential heterogeneity of variances between the treatment groups. Following the Welch ANOVA, we performed individual t-tests at specific time intervals to explore where statistically significant differences existed. The Bonferroni correction was applied to adjust for multiple comparisons, reducing the risk of Type I errors (false positives). To further investigate the differences in the percentage of chemicals leaching over time within each treatment group while accounting for the repeated measurements, a Tukey test was conducted. This test allowed us to identify specific time points where significant differences in chemical leaching occurred, providing a more comprehensive understanding of the temporal dynamics within each treatment group.

All statistical analyses were conducted using JMP Pro 16 (JMP Software), and significance levels were set at p < 0.05.

## Results and discussion

### PCB sorption onto HDPE and PVC

To confirm our estimated amount of plastic-sorbed chemicals, plastic pieces were incubated for 20h in hexane. A maximum amount of 43.1 ng PCB-28 and 38.1 ng PCB-138 per plastic piece was expected to be measured if all the PCBs had homogeneously sorbed at the surface of plastic. We found 42.3 ± 1.4 ng PCB-28/piece of HDPE, 35.6 ± 3.3 ng PCB-138/piece of HDPE, 42.1 ± 8.1 ng PCB-28/piece of PVC and 35.8 ± 5.6 ng PCB-138/piece of PVC leached from the plastic in hexane (S2 Fig). Negligible, non-quantifiable amounts of PCBs were detected in salt water after the 3-week incubation concluding that all PCBs were sorbed onto plastics after 3 weeks, without distinction between the type of polymer (S2 Fig). Importantly, no trace of PBDE-209 were measured in salt water after 3 weeks, suggesting that no loss of PBDE-209 from plastics occurred during the PCB sorption experiment.

### Influence of food and gastric secretions on chemical leaching

#### Oil versus aqueous solution

**Fig 2:**
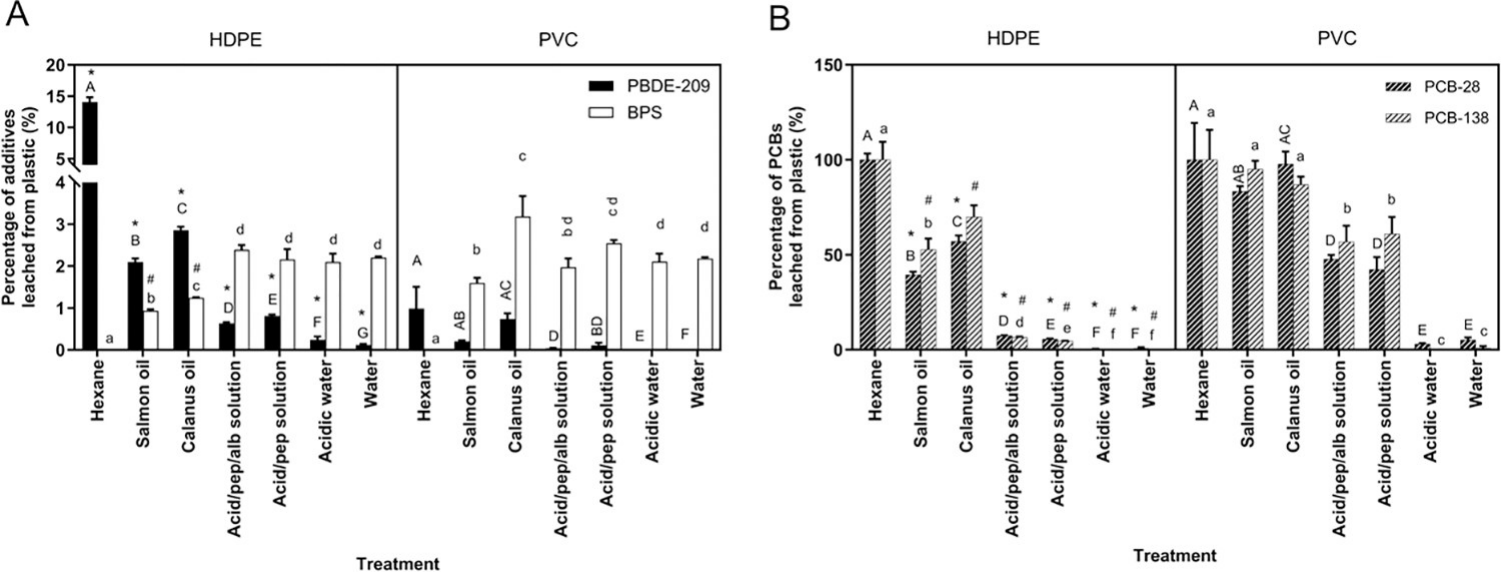
Percentage of plastic additives (A) and PCBs (B) released from HDPE and PVC in different solutions after 20h. HDPE and PVC pieces with 1% PBDE-209 + PCB-28 and -138 or HDPE and PVC pieces with 1% BPS were leached in hexane, salmon oil, calanus oil, an aqueous digestive solution made of albumin, pepsin, HCl and water (“Acid/pep/alb solution”), an acidic pepsin solution (“Acid/pep solution”), acidic water and water for 20h at 38°C (800 rpm; n = 3). Panel A) the concentrations of additives (PBDE-209 in black and BPS in white) leached from HDPE and PVC. Panel B) the concentrations of PCBs (PCB-28 in black and PCB-138 in white) leached from HDPE and PVC. Values with different uppercase letters (A, B, C, D, E, F for PBDE-209 or PCB-28) or lowercase letters (a, b, c, d, e, f for BPS or PCB-138) show significant differences between treatments (p-val < 0.05). Asterisk (*) (PBDE-209 or PCB-28) and pound sign (#) (BPS or PCB-138) depict significant differences between polymers for each chemical and treatment (p-val < 0.05). Error bars represent standard deviation, n = 3.

*PBDE-209*, *PCB-28 and—138*. For both polymer types, PBDE-209 and PCBs were leached at significantly higher proportions in oil than in an aqueous solution (acidic albumin-pepsin solution, acidic pepsin solution, acidic water and water) (Fig 2). For HDPE compared to the acidic albumin-pepsin solution, the leaching of PBDE-209 was 3.3-fold higher in salmon oil and 4.5-fold higher in calanus oil (Fig 2A), the leaching of PCB-28 was 5.3-fold higher in salmon oil and 7.6-fold higher in calanus oil (Fig 2B), and the leaching of PCB-138 was 8.8-fold higher in salmon oil and 10.7-fold higher in calanus oil (Fig 2B). For PVC compared to the acidic albumin-pepsin solution, the leaching of PBDE-209 was 5.2-fold higher in salmon oil and 18.4-fold higher in calanus oil (Fig 2A) and the leaching of PCB-28 and PCB-138 was about twice as high in both oils (Fig 2B). In the complete PGM, including both oil and aqueous solution, PBDE-209 and PCBs were only detected in salmon or calanus oil, confirming their high affinity for lipids, which may be explained by the hydrophobicity of these chemicals, illustrated by high log K_ow_ of 9.97 [39], 5.67 [62] and 6.83 [62] for PBDE-209, PCB-28 and PCB-138 respectively. Our results are consistent with other *in vitro* studies in seabirds showing that stomach oil significantly increases the leaching of PBDE-209 from plastics compared to water-based solutions such as distilled water, seawater, and an acidic pepsin solution [20, 22].

*BPS*. The leaching of BPS from HDPE followed opposite trends compared to PBDE-209, with significantly higher concentrations leached in the aqueous solutions compared to the oils (Fig 2A). More specifically, the leaching of BPS from HDPE was 2.6-fold higher in the acidic albumin-pepsin solution than in salmon oil and 1.9-fold higher in the acidic albumin-pepsin solution than in calanus oil. The same trend was observed for HDPE in the complete PGM where higher concentrations of BPS (1.2 to 1.7 times) were found in the aqueous phase of the gastric solution compared to the lipid phase (salmon or calanus oil) (S3 Fig). This higher affinity of BPS for aqueous environments compared to PBDE-209 is consistent with the polar sulfonyl groups of BPS, which significantly increase its solubility in water (3518 mg/L), as illustrated by the lower log K_ow_ of the molecule (1.65) [42]. Its positive log Kow and ability to create hydrophobic interactions explain why it is also found in the lipid compartment [63]. Surprisingly, the leaching of BPS from PVC did not support this pattern. When gastric components were tested individually, a higher (1.6-fold) proportion was leached in the calanus oil than in the aqueous solutions, followed by a lower (1.2-fold) proportion in the salmon oil than in the aqueous solutions (Fig 2A). In the PGM, a higher proportion of BPS was also found in the calanus lipid phase compared to the aqueous phase during the first 20 hours, whereas no difference was found in the distribution of BPS between salmon lipid and aqueous phases (S3 Fig). These observations can partly be attributed to the distribution of BPS within the polymer matrices and the stronger influence of WEs on the leaching of chemicals (discussed below).

Overall, our findings indicate that the release of highly hydrophobic chemicals, such as PBDEs and PCBs, from plastics within the Procellariiform stomach is primarily influenced by the presence of lipids. In contrast, the leaching of more water-soluble additives, like BPS, is affected by both lipids and aqueous components, with an affinity for one or the other depending on whether the oil and aqueous components are combined, the type of lipid involved and the type of polymer.

#### Calanus versus salmon oil

**Fig 3:**
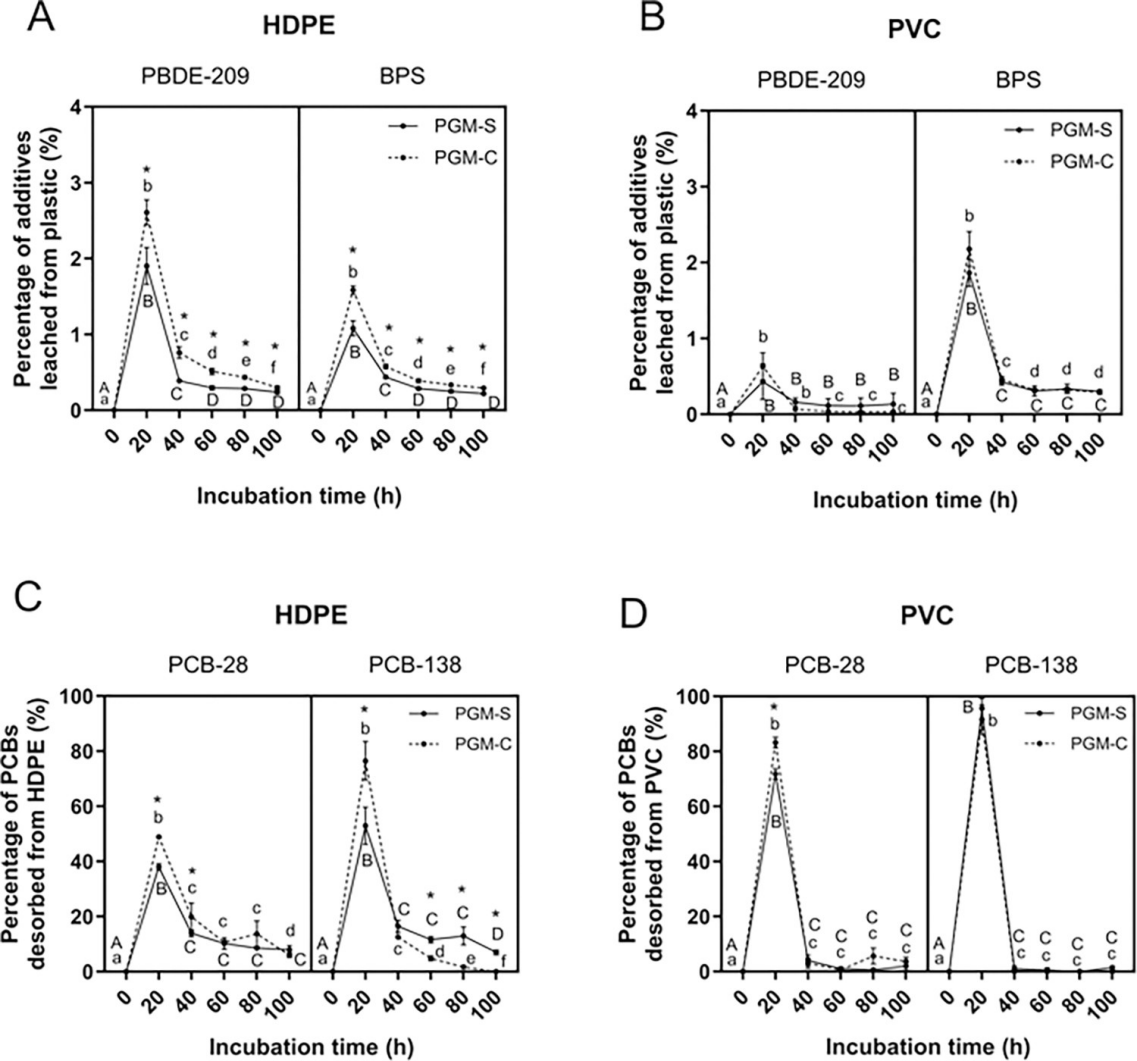
Percentage of plastic additives and PCBs released from HDPE and PVC in salmon or calanus gastric fluid over time. HDPE and PVC pieces with 1% PBDE-209 + PCB-28 and -138 or HDPE and PVC pieces with 1% BPS were leached in the full PGM containing an acidic albumin-pepsin solution (pH 3) mixed with salmon oil (PGM-S) or calanus oil (PGM-C) at 38°C for 100h (800 rpm; n = 3). The mixture was renewed every 20h. Aqueous and lipidic phases were analyzed separately and concentrations were summed to give the total concentration leached in the digestive mixtures. Concentrations were below the detection limit in the aqueous phases for PBDE-209 and PCBs. Panel A and B) the percentage of PBDE-209 and BPS leached from HDPE and PVC. Panel C and D) the percentage of PCB-28 and PCB-138 desorbed from HDPE and PVC. Values with different uppercase letters (A, B, C, D, E) and lowercase (a, b, c, d, e) show significant differences in the percentage of chemicals released (additives or PCBs) over time for each treatment (p-val < 0.05). Asterisk symbols (*) show significant differences between salmon and calanus gastric fluid for each chemical at each timepoint (p-val < 0.05). Error bars represent standard deviation, n = 3.

The lipid composition of the diet (calanus or salmon oil) had a significant effect on the leaching of plastic-associated compounds with more additives and PCBs leached in calanus oil compared to salmon oil. For HDPE, calanus oil contained 1.3-fold more BPS and PCB-138 and 1.4-fold more PBDE-209 and PCB-28 than salmon oil (Fig 2A and 2B). For PVC, calanus oil contained 3.6-fold more PBDE-209, 3.9-fold more BPS and 1.2-fold more PCB-28 than salmon oil (Fig 2A and 2B). PCB-138 was entirely leached from PVC in both oils after 20h (Fig 2B). The observed differential influence between oils can be in part explained by the lipid composition of salmon versus calanus oil. Salmon oil is primarily made of TAGs (> 90%) while calanus oil is primarily made of WEs (> 90%) (S1 Table and S4 Fig). As such, salmon oil is characterized by a higher concentration of polyunsaturated fatty acids (PUFAs) whereas calanus oil contains more long-chain monounsaturated fatty acids and long-chain monounsaturated fatty alcohols, potentially creating a greater affinity for hydrophobic chemicals. Longer alkyl chains or lower number of unsaturated sites have been related to a higher hydrophobicity of fatty acids and fatty alcohols [64]. When considering the complete PGM, a similar trend was observed for HDPE (PGM-C contained levels of PBDE-209, BPS, PCB-28 and -138 that were 1.4-, 1.5-, 1.3- and 1.4-fold higher than in PGM-S) (Fig 3A and 3C). It was also observed for the leaching of PCB-28 from PVC (1.5-fold higher in PGM-C than in PGM-S) (Fig 3D). On the other hand, the leaching of PBDE-209, BPS and PCB-138 from PVC did not differ between PGM-S and PGM-C (Fig 3B and 3D), which can be attributed to the differential leaching behavior of chemicals between polymers (discussed in the section “Influence of the type of polymer on chemical leaching”).

Procellariiform stomach oil is mainly made of TAGs and WE (70–90% total lipids) [36, 37, 57]. Connan et al. (2007) observed that TAG/WE ratio in stomach oil could be very different depending on the Procellariiform species, ranging from rich-WE and poor-TAG oil (short-tailed shearwater *Puffinus tenuirostris* and thin-billed prion *Pachyptila belcheri*) to rich-TAG and poor-WE oils (white-chinned petrel *Procellaria aequinoctialis*) with an intermediate group (Antarctic prion *Pachyptila desolata* and blue petrel *Halobaena caerulea*) varying with the dietary habits [38]. The consumption of fish and squid will mainly provide TAGs while the consumption of crustaceans (krill, copepods) will mainly provide WEs [38]. Accordingly, our data suggest a richer crustacean diet may result in a higher proportion of chemicals leached from ingested plastics in a Procellariiform’s stomach.

#### Acidity, enzymes and proteins

*PBDE-209*, *PCB-28 and—138*. Our results showed that acidity (HCl, pH 3) did not affect the leaching of PCBs from either HDPE or PVC (Fig 2B) and only slightly increased the leaching of PBDE-209 from HDPE (Fig 2A). However, the addition of an enzyme (pepsin) in solution with acidic water (pH 3) significantly increased the leaching of PBDE-209 and PCBs from both polymers as compared to acidic water or water alone (Fig 2A and 2B). For HDPE, the leaching of PBDE-209 and PCB-28 was 3.3 and 13.6-fold higher in the acidic-pepsin solution compared to acidic water. For PCB-138, 4.6% were leached from HDPE in the acidic-pepsin solution while nothing was detected in acidic water (Fig 2B). For PVC, 0.11% PBDE-209 were leached in the acidic-pepsin solution while nothing was detected in acidic water (Fig 2A). The addition of pepsin had a particularly strong effect on the leaching of PCBs from PVC, where 60.9% PCB-138 were leached in acidic-pepsin solution while nothing was detected in acidic water (Fig 2B). For PCB-28, the leaching from PVC was 13.8-fold higher in the acidic pepsin-solution (Fig 2B). Our results are consistent with the observation made by Tanaka et al. (2015) where PBDE-209 leaching in the presence of pepsin acidic water was higher than in seawater or distilled water [20].

Proteins such as pepsin are known to form coronas at the surface of different types of structures including plastics [65]. PCB desorption in the presence of pepsin may have been driven by the surface-active behavior of the proteins, leading to a competition for the interface between both PCBs and the proteins, displacing the compound previously adsorbed [66]. The higher PCB desorption from PVC in the presence of pepsin was likely due to its glassy characteristic (discussed in the next section). The addition of albumin to the acidic pepsin solution slightly reduced the leaching of PBDE-209 from HDPE by a factor of 1.3 (Fig 2A) and slightly increase the leaching of PCB-28 and -138 by a factor of 1.3 and 1.4 respectively (Fig 2B). The presence of albumin did not further influence the leaching of plastic-associated chemicals from PVC.

*BPS*. In general, BPS leaching was not influenced by the pH or the presence of pepsin and albumin, showing percentages of leaching similar to those found in water alone (about 2%; Fig 2A). It is possible that BPS’s affinity for water dominated and concealed the lesser effects of pH, pepsin or albumin.

### Influence of the type of polymer on chemical leaching

Polyethylene (PE) is the most abundant polymer found to be ingested by Procellariiforms, followed by polypropylene (PP) and PVC [67]. We used HDPE, a rubbery semi-crystalline polymer similar to PP and characterized by a higher free volume and mobility as well as PVC, a glassy amorphous polymer characterized by a denser and more compact structure [29], in our gastric model to assess exposure risk to plastic-associated chemicals. We found that the proportion of chemicals leached from plastic was dependent on the type of polymer (HDPE versus PVC).

#### PBDE-209

Significantly less PBDE-209 was leached from PVC compared to HDPE, ranging from ~ 4-fold less in calanus oil alone (Fig 2A) and the PGM (first 20h) (Fig 3A) to 15.9-fold less in acidic albumin-pepsin solution alone (Fig 2A). The polymer-dependent differences in chemical leaching might be explained by the additive’s distribution within the polymer matrices as well as by the polymer physico-chemical properties. Despite the homogenization step performed during plastic manufacture to obtain a homogeneous additive distribution, surface tension-driven migrations may possibly occur upon molding, leading to heterogeneity in the final plastic plates. Time-of-Flight Secondary Ion Mass Spectrometry (ToF-SIMS) analyses performed using the depth-profiling mode revealed that PBDE-209 was enriched on the outermost surface of HDPE pieces, but not PVC (S1 Fig), likely resulting in a higher percentage of PBDE-209 leached from HDPE. In addition, rubbery polymers such as HDPE are expected to demonstrate a great diffusion potential due to their large free volume and great flexibility and molecular mobility [29]. Conversely, the large size and high molecular mass of PBDE-209 (959.17 g/mol) may have reduced its ability to diffuse out of the more compact PVC matrix.

#### BPS

The opposite was observed for the leaching of BPS in salmon and calanus oils, in which it was 1.7 and 2.6 times more leached from PVC than from HDPE (Fig 2A). A similar trend was also observed when the oil was mixed with the aqueous solution (PGM-S and -C), where ~1.5 times more BPS leached from PVC than from HDPE over the first 20h (Fig 3A and 3B). ToF-SIMS analysis revealed that BPS was enriched in the superficial layers of PVC, while mostly absent from the surface and subsurface layers of HDPE, which may explain the larger quantities leached from PVC (S1 Fig). In addition, the smaller size and lower molecular mass (250.28 g/mol) of BPS also facilitates its ability to diffuse out of the PVC matrix. On the other hand, no difference was observed between PVC and HDPE in the leaching of BPS in the different aqueous solutions. This lack of distinction may be explained by the pronounced affinity of BPS for water, which may have masked differences between polymers (Fig 2A).

The affinity of BPS for oils and aqueous environments was polymer-dependent. As previously discussed, its leaching from HDPE was greater in the aqueous environment than in lipids (calanus oil > salmon oil) (Fig 2A), while its leaching from PVC varied depending on whether the oil and aqueous solutions were tested individually (calanus oil > aqueous solutions > salmon oil) (Fig 2A) or in combination [PGM-S = PGM-C (Fig 3B) with calanus oil > aqueous phase = salmon oil during the first 20 hours (S1 Fig)]. Our findings indicate that BPS may exhibit a stronger affinity for calanus oil, rich in wax esters, compared to a water-rich medium when its leaching is facilitated by a higher distribution at the superficial layers of the plastic. However, this affinity may be masked in the presence of aqueous components (Fig 3B and Table 1). To our knowledge, no study has reported the variation in affinity of BPS or its analogs for a lipid or aqueous environment as a function of the specific type of polymer and lipids involved. This observation warrants further investigation.

**Table 1:** Total percentage (%) and concentration (μg/g) of PBDE-209, BPS, PCB-28 and PCB-138 leached after 100 hours in the PGM.

Polymer	Treatment	Chemical leached							
		PBDE-209		BPS		PCB-28		PCB-138	
		% ± SD	[μg/g] ± SD	% ± SD	[μg/g] ± SD	% ± SD	[μg/g] ± SD	% ± SD	[μg/g] ± SD
HDPE	PGM-S	3.1 ± 0.3	311.2 ± 28.2	2.3 ± 0.07	226.9 ± 6.6	78.6 ± 2.5	1.3 ± 0.07	100.9 ±10.0	1.4 ± 0.17
	PGM-C	4.6 ± 0.3	461.0 ± 30.2	3.2 ± 0.02	317.4 ± 2.1	99.0 ± 3.2	1.7 ± 0.17	107.1 ± 10.6	1.5 ± 0.39
PVC	PGM-S	0.9 ± 0.4	94.6 ± 43.3	3.2 ± 0.29	323.1 ± 29.4	79.5 ± 16.0	0.9 ± 0.02	98.7 ± 15.3	0.9 ± 0.05
	PGMC	0.8 ± 0.2	80.0 ± 18.4	3.6 ± 0.17	357.1 ± 17.1	96.1 ± 19.3	1.0 ± 0.05	91.5 ± 14.2	0.8 ± 0.05

HDPE and PVC pieces with 1% PBDE-209 + PCB-28 and -138 (sorbed) or HDPE and PVC pieces with 1% BPS were leached in the complete PGM containing an acidic albumin-pepsin solution (pH 3) mixed with salmon oil (PGM-S) or calanus oil (PGM-C) at 38°C for 100h (800 rpm; n = 3). The total percentage (%) or concentration (μg/g) of chemical leached after 100 hours were measured by summing the percentage or concentration of chemicals leached from each polymer after 20, 40, 60, 80 and 100 hours in the PGM-S or PGM-C.

#### PCB-28 and -138 (sorbed)

A higher proportion of both PCB-28 (1.7 to 6.4-fold) and -138 (1.8 to 8.7-fold) was leached from PVC compared to HPDE in oil as well as aqueous solutions after 20h. The higher free volume and diffusivity of HDPE versus PVC resulted in sorption of chemicals deeper into the plastic matrix [68], which in turn, is associated with a slower leaching of chemicals during leaching processes.

### Influence of contact time in the PGM for chemical leaching

During digestion, the digestive fluids of a Procellariform stomach can be divided into two phases: a lipidic phase (stomach oil) and an aqueous phase (water, HCl, enzymes and proteins) [50]. Plastics trapped in the gizzard (second stomach chamber) are exposed to both phases in addition to undergoing mechanical digestion. Because both lipidic and aqueous elements influence the leaching of chemicals [20, 69], plastic pieces were incubated in the PGM combining the lipidic and aqueous phases [acidic albumin-pepsin solution mixed with salmon (PGM-S) or calanus (PGM-C) oil] for 100 hours.

We found a peak in the leaching of chemicals during the first 20h followed by a significant decrease for the remaining 80 hours (Fig 3).

#### PBDE-209 and BPS (additives)

For HDPE, 1.9 ± 0.2% and 2.6 ± 0.2% of PBDE-209 were leached in PGM-S and PGM-C respectively, during the first 20h, while only 0.4 ± 0.03% and 0.8 ± 0.1% were leached between 20 and 40 hours (Fig 3A). The percentage leached continued to decrease slowly but significantly until the end of the experiment (100 hours). A similar dynamic was observed for BPS, with a leaching of 1.8 ± 0.1% and 1.6 ± 1.0% in PGM-S and PGM-C, during the first 20 hours, followed by 0.4 ± 0.02% and 0.6 ± 0.03% between 20 and 40 hours (Fig 3A).

For PVC, the peak leaching of PBDE-209 was lower than HDPE, with 0.4 ± 0.2% and 0.6 ± 0.2% of PBDE-209 leached in PGM-S and PGM-C during the first 20 hours of contact (Fig 3B). A significant drop in the proportions leached was observed between 20 and 40 hours, with 0.2 ± 0.1% and 0.1 ± 0.01% of PBDE-209 in PGM-S and PGM-C. BPS was leached at 1.9 ± 0.2% and 2.2 ± 0.2% in PGM-S and PGM-C over the first 20 hours before dropping at 0.4 ± 0.04% and 0.5 ± 0.04% between 20 and 40 hours (Fig 3B). In both cases, levels continued to drop between 40 and 60 hours and then remained constant.

The higher proportion of additives leached during the first 20 hours of digestion (Fig 3A and 3B) could be attributed to the higher leachability of additives in the superficial layers of the plastic, which are readily accessible to the surrounding solution. The heterogeneous distribution of additives within the polymer matrices identified by ToF-SIMS support this observation; higher proportions of PBDE-209 and BPS were present in the superficial layers of HDPE and PVC respectively. These observations are consistent with other studies showing an exponential decrease in the leaching of additives from PE in oil and water over time [20, 69].

#### PCB-28 and -138 (sorbed)

The proportion of PCBs leached from HDPE was much higher than the one of PBDE-209 and BPS, with peaks of 38.0 ± 1.2% and 48.9 ± 0.3% of PCB-28 and 52.9 ± 6.7% and 76.5 ± 7.0% of PCB-138 leached in PGM-S and PGM-C during the first 20 hours of contact (Fig 3C). The proportions then dropped to 13.9 ± 1.3% and 19.9 ± 5.0% of PCB-28 and 16.4 ± 2.2% and 12.6 ± 0.2% of PCB-138 between 20 and 40 hours. Levels continued to decline over time until the end of the experiment (100 hours). PCBs were also leached from PVC in much higher proportions than PBDE-209 and BPS with peaks of 71.8 ± 1.9% and 48.9 ± 0.3% of PCB-28 and 95.6 ± 3.7% and 91.5 ± 5.4% of PCB-138 in salmon and calanus gastric solution, respectively (Fig 3D). Proportions leached then dramatically dropped between 20 and 40 hours of contact, with levels below 1%.

#### Additives versus sorbed PCBs

The majority (79–100%) of sorbed PCBs were leached after 100 hours while only a small percentage of additives leached out (less than 5%). When considering total μg/g, 100–450 times more additives were leached compared to PCBs (Table 1) which can be attributed to the higher concentration of additives present within the plastic (10,000 μg additive/g of plastic versus 925–1700 μg PCBs/g plastic)–which is representative of what happens in the environment [49]. Our findings suggest that after ~ 5 days in a Procellariiform stomach, plastics could leach all the sorbed chemicals, but a substantial amount of additives remain in the plastic matrix to potentially leach in the following days or weeks.

### Implications of plastic ingestion on seabird’s health

Out of 226 seabird species studied for plastic ingestion, 80% have been found with plastics in their stomach, with Procellariforms accounting for half of this percentage [1]. In addition to their surface-feeding behavior making them more likely to ingest floating plastics than diving seabird species [59, 70, 71], Procellariiforms rarely regurgitate indigestible items, which increases their propensity to accumulate plastics in their stomach for extended periods of time [27].

Our study explores the multifaceted interactions between polymer type, gastric content, retention time and chemical properties of plastic-associated chemicals (additives and sorbed POPs) on their leaching from plastics under Procellariform gastric conditions. A comprehensive overview of experimental factors can be found in Table 2. Our results showed a strong influence of the type of polymer on the leaching behavior of chemicals, since higher proportions of PBDE-209 were leached from HDPE while higher proportions of PCBs and BPS were leached from PVC, which can be attributed to the chemical properties of plastic-associated compounds, their distribution within the polymer matrix as well as the diffusibility of the polymer. For BPS, this difference between polymer type was only observed in the presence of lipids (alone or in the PGM), also highlighting the strong influence of lipids on the leaching behavior of chemicals. The leaching of highly hydrophobic chemicals (PBDE-209 and PCBs) was primarily enhanced by the presence of oils. However, the behavior of BPS was more complex, as its affinity for lipids depended on the type of polymer. When leached from HDPE, BPS exhibited a stronger affinity for the aqueous environment (alone or in the PGM). However, when leached from PVC, calanus oil (alone or in the PGM-C) had a stronger impact than the aqueous environment, which can be partly attributed to the wide distribution of BPS at the surface of PVC and the stronger influence of calanus wax ester-rich oil on the release of plastic-associated chemicals. Indeed, the presence of calanus oil almost always resulted in higher proportions of leached chemicals compared to salmon oil, highlighting the potentially stronger influence of a WEs-rich diet compared to TAGs-rich diet on the leaching of plastic-associated chemicals. It should be noted that the influence of calanus oil was nuanced by the type of polymer and the presence of aqueous components.

**Table 2:** Comprehensive overview of the influence of the polymer type and digestive conditions on the leaching of PBDE-209, PCBs and BPS.

	*Influence type of polymer*				
	**HDPE**			**PVC**	
	**Individual**		**PGM**	**Individual**	**PGM**
PBDE-209	+++		+++	+	+
PCBs	+		+	+++	+++
BPS	Oils +		+ During first 20 hours	Oils +++	+++ During first 20 hours
	Aqueous solutions =			Aqueous solutions =	
	***Influence lipidic and aqueous environment***				
	**HDPE**			**PVC**	
	**Individual**		**PGM**	**Individual**	**PGM**
PBDE-209	Lipidic > aqueous environment		Only found in lipidic phase	Lipidic > aqueous environment	Only found in lipidic phase
PCBs	Lipidic > aqueous environment		Only found in lipidic phase	Lipidic > aqueous environment	Only found in lipidic phase
BPS	Aqueous > lipidic environment		Found in both phases Aqueous > lipidic phase	Calanus oil > Aqueous solution > Salmon oil	Found in both phases PGM-C: Lipidic > aqueous phase during first 20 hours PGM-S: Lipidic = aqueous phase
	***Influence type of oil***				
	**HDPE**			**PVC**	
	**Individual**	**PGM**		**Individual**	**PGM**
PBDE-209	Calanus oil > salmon oil		PGM-C > PGM-S	Calanus oil > salmon oil	PGM-C = PGM-S
PCBs	Calanus oil > salmon oil		PGM-C > PGM-S	Calanus oil > salmon oil	PCB-28: PGM-C > PGM-S first 20 hours PCB-138: PGM-C = PGM-S
BPS	Calanus oil > salmon oil		PGM-C > PGM-S	Calanus oil > salmon oil	PGM-C = PGM-S

Three questions are summarized in this table: the influence of the polymer type, the influence of lipids compared to the aqueous environment, and the influence of the type of oil on the leaching of plastic-associated chemicals. The table is divided according to the type of polymer involved, and whether the oil and aqueous components were studied individually (noted as “individual”) or combined (noted as “PGM”). Higher, lower or similar proportions of pollutants leached from one polymer compared to another is demonstrated by "+++", "+" or “=“ respectively.

PCBs were completely leached after 100 hours in the PGM. However, in the environment, it is possible that POPs may leach over longer periods of time. Indeed, plastics in sea water can be in contact with POPs for months or even years, which can lead to a deeper sorption of POPs inside the plastic at equilibrium [12, 72]. A deeper sorption may in turn lead to a slower release in the digestive tract. Regardless of the desorption time, concentrations of PCBs leached from the plastic are marginal compared to the exposure resulting from POPs that bioaccumulate in bird prey [73], highlighting the negligible impact of sorbed POPs compared with other natural pathways of exposure. On the other hand, the continued leaching of additives was a concerning finding. Only a small fraction of additives (< 5%) was leached out during the residence time in the artificial stomach, suggesting that the remaining > 95% could continue to leach over time, subjecting Procellariiform to chronic exposure to these chemicals for weeks or even months. Since additives are present at considerably higher concentrations than sorbed contaminants (0.05–70% of additive per plastic weight [44] compared to on average < 0.003% of POP per plastic weight [74]), they could be released at levels thousand times higher than sorbed POPs. Moreover, many plastic additives are non-persistent pollutants (e.g. bisphenol A and its analogous, phthalates, alkylphenols) which are found at higher concentrations in plastic than in natural prey [75]. Seabird exposure to this type of molecule is therefore mainly due to plastic ingestion, unlike exposure to POPs. This chronic exposure to plastic-associated chemicals raises concerns about potential health risks to seabirds, as most of these compounds are known as endocrine disruptors [76–79]. Indeed, many plastic additives have been associated with negative reproductive effects such as delays in sexual maturity, decrease of fertility, gonadal abnormalities (feminization/masculinization) and development of cancer [19, 76–78].

In conclusion, our study underscores that plastic ingestion by seabirds may pose a substantial health risk, primarily due to the leaching of significant concentrations of additives. Based on these findings, future research should prioritize investigating the potential health effects arising from the release of these plastic additives when ingested by seabirds.

## Supporting information

S1 Protocol Chemicals and materials. (PDF)

S2 Protocol Plastic manufacture. (PDF)

S3 Protocol Additional information on chemical analysis. (PDF)

S4 Protocol Statistical analysis. (PDF)

S1 Fig Intensity of selected negatively-charged secondary ions recorded using ToF-SIMS upon depth-profiling on a) HDPE + PBDE209; b) PVC +PBDE209; c) HDPE + BPS and d) PVC + BPS. (PDF)

S2 Fig Desorption of PCBs from HDPE and PVC in hexane after 20h. (PDF)

S3 Fig Concentration of BPS released from HDPE (1) and PVC (2) in each phase of the salmon or calanus gastric fluid over time. (PDF)

S4 Fig Example of wax ester found in the oil of calanus finmarchicus. (PDF)

S1 Table Lipidic composition of the salmon and calanus oil. (PDF)
