# Supplementary material for: Dynamics of leaching of POPs and additives from plastic in a Procellariiform gastric model: Diet- and polymer-dependent effects and implications for long-term exposure
Source: PLoS One. 2024 Mar 27;19(3):e0299860. doi: 10.1371/journal.pone.0299860 (PMC10971572; doi:10.1371/journal.pone.0299860)
Supplement: S1 Protocol — (PDF) [file pone.0299860.s001.pdf]

S1 Protocol. Chemicals and materials

High density polyethylene (HDPE) pellets (Hostalen GC7260LS from) were purchased from Lyondellbasell (Brussels, Belgium) and polyvinylchloride (PVC) pellets (ER 027/W141/AC) were purchased from Bevnic (Dijon, France). Analytical-grade polybrominated diphenyl ether-209 (PBDE-209), polychlorinated biphenyl -28 and -138 (PCB-28 and PCB-138) and bisphenol-S (PBS) and bisphenol S-d_8 (BPS-d_8) were purchased from LGC Standards (Molsheim, France). Beaphar® Salmon oil was purchased from Aveve (Wavre, Belgium). Calanus® Oil was obtained from Zooca™ (Tromsø, Norway). NaCl and Pepsin A were purchased from Sigma-Aldrich (Overijse, Belgium). Albumin (V fraction) was purchased from Carl Roth® (Karlsruhe, Germany). Analytical-grade hydrochloric acid 37%, hexane and methanol were purchased from VWR™ (United Kingdom).

Glass bottles (500 mL) were used to incubate plastic samples in saltwater spiked with PCBs. Glass Erlenmeyers (50 mL) sealed with foil-lined rubber caps and parafilm were used to incubate plastic in gastric solutions, hexane and water. Round glass stirring bars used to mix the digestive solutions were purchased from Sigma-Aldrich (Overijse, Belgium). Samples were collected with cotton-plugged Pasteur pipets and stored in glass vials with polypropylene caps lined with aluminum foil.
