# Supplementary material for: Dynamics of leaching of POPs and additives from plastic in a Procellariiform gastric model: Diet- and polymer-dependent effects and implications for long-term exposure
Source: PLoS One. 2024 Mar 27;19(3):e0299860. doi: 10.1371/journal.pone.0299860 (PMC10971572; doi:10.1371/journal.pone.0299860)
Supplement: S2 Protocol — (PDF) [file pone.0299860.s002.pdf]

**S2 Protocol. Plastic manufacture**

HDPE (Hostalen GC7260LS from Lyondellbasell (Brussels, Belgium)) or PVC (ER 027/W141/AC) from Bevnic (Dijon, France)) pellets were melted at 190°C and 170°C respectively for 1 minute in a monitor mixer containing two screws to ensure homogenization when rotating. One percent of additive (PBDE-209 or BPS; powder form) were added and mixed with the rest for 2 more minutes. Following that, the material was placed into a 1 mm thick frame, between 2 stainless steel plates coated with non-stick paper impregnated with Teflon and put into a fusing press at 190°C or 170°C for 5 minutes. After being cooled down to 40°C at 15°C/minute, the frame – steel plate – Teflon  paper – plastic system was removed from the press and the plastic plate was recovered.
