# Supplementary material for: Dynamics of leaching of POPs and additives from plastic in a Procellariiform gastric model: Diet- and polymer-dependent effects and implications for long-term exposure
Source: PLoS One. 2024 Mar 27;19(3):e0299860. doi: 10.1371/journal.pone.0299860 (PMC10971572; doi:10.1371/journal.pone.0299860)
Supplement: S3 Protocol — (PDF) [file pone.0299860.s003.pdf]

S3 Protocol. Additional information on chemical analysis

S3.1. PBDE-209 and PCB analysis

Solid-liquid adsorption chromatography

According to the Beltest I014 method[1], silica gel column composed of 0.5 g of anhydrous Na_2SO_4, 1 g of Al_2O_3 containing 11% of water, 6 g of acidified silica gel (60/40 silica/H_2SO_4) and, on the top, 0.5 g of anhydrous Na_2SO_4 were conditioned with 40 ml of n-hexane. Solutions were then loaded onto the columns and rinsed three times with 5 ml of hexane. The column was further eluted with 20 ml of n-hexane.

GC/HRMS analysis

Analyses were performed on an Autospec Ultima mass spectrometer (Waters, Manchester,UK) coupled to an Agilent 6890 gas chromatograph (Palo Alto, CA) equipped with a splitless and a Programmable Temperature Vaporisation (PTV) injection port. For PCBs, 2 µL of samples were injected at 275°C in splitless mode on an HT8 (25m x 0.22mm x 0.25µm ; SGE) at a 0.8 mL/min He (6.0 purity ; Air Liquide) constant flow with the following temperature ramp: 140°C for 2 min, heated to 220°C at 15°C/min and held for 7.5 min, followed by an increase to 250°C at 6°C/min, to 265°C at 2°C/min and finally to 310°C at 28°C/min kept for 0.7 min. LOD and LOQ were 0.27 and 0.82 ng/ml for PCB-28 and 0.03 and 0.10 ng/mL for PCB-138. For BDE-209, 5 µL of samples were injected at 80°C in PTV mode on an RTX-1614 (15m x 0.25mm x 0.10µm; Restek) at a 1.5 mL/min He constant flow with the following temperature program: 120°C held for 2 min followed by a 15°C/min increase to 205°C and a 6°C/min to 315°C kept for 1 min. LOD was 0.003 ng/mL LOQ was 0.01ng/mL. Both analyses were performed in Selected Ion Monitoring (SIM) at 10,000 mass resolution with Electronic Ionization at 35 eV. Two ions (quantifier and qualifier) were recorded for each analyte and their isotopic ion ratio was checked for interferences. The quantitative aspect was achieved through the isotope dilution technique using ^{13}C labelled analogous of each analyte.

S3.2. BPS analysis

HPLC-MS analysis

An Agilent 6050B series single quadrupole LC/MS-ESI system equipped with an Agilent 1290 infinity UHPLC system was used for analysis. The chromatographic separation was carried out on an Agilent column RRHD Eclipse plus C18 (100 mm X 2.1 mm, 1.8 µm) with an Agilent Eclipse C18 (5mm x 2.1mm, 1.8µm) guard column. All R^2 values of standard linear regression curves of measured BPS were > 0.99. For BPS, LOD was 0.033 µg/mL and LOQ was 0.125 µg/mL. For BPS-d8, LOD was 0.036 µg/mL and LOQ was 0.133 µg/mL. Samples were analyzed in negative ionization mode. Under single ion monitoring (SIM), a m/z of 250 was used for BPS analysis $[MM+H]^+$ and a m/z of 258 for BPS-d8.The mobile phase consisted of milli-Q water: acetonitrile (95:5) with 0.1% of formic acid (A) and acetonitrile: milli-Q water (95:5) with 0.1% of formic acid (B) and the flow rate was 0.3 mL/min. The gradient elution program was performed as follows: 0.0 – 1.0 min, 95% A – 5% B; 1.0 min – 7.0 min, 0% A – 100%; 7.0 – 9.0 min, 0% A – 100% B; 9.0 – 10.0 min, 95% A – 5% B; 10.0 – 12.0 min, 95% A – 5% B (total run time 12 minutes). The injection volume of the sample solution was 5 µL. The temperature of the column was controlled at 40°C. Capillary voltage was set to 3.1 kV, nozzle voltage was set to 400 V (negative) and 1000 V

(positive). Sheath gas temperature was 125°C, drying gas temperature was 250°C and drying gas flow was 9 L/min. The fragmentor was set to 90 eV.

References

1. Beltest I014-Rev3 (2000) Determination of polychlorinated biphenyls in animal feed, animal fat, egg and egg products, milk products and other foodstuffs. Beltest, Belgium.
