# Supplementary material for: Dynamics of leaching of POPs and additives from plastic in a Procellariiform gastric model: Diet- and polymer-dependent effects and implications for long-term exposure
Source: PLoS One. 2024 Mar 27;19(3):e0299860. doi: 10.1371/journal.pone.0299860 (PMC10971572; doi:10.1371/journal.pone.0299860)
Supplement: S4 Protocol — (PDF) [file pone.0299860.s004.pdf]

S4 Protocol. **Statistical analysis**

Percentage of additive leached in each solution

The percentage of additive desorbed in each solution was calculated as follow:

$$Percentage\ additives\ leached\ (\%) = \frac{\left(\frac{conc. sample\ 1\ \left(\mu\frac{g}{g}\right) +\ conc. sample\ 2\ \left(\mu\frac{g}{g}\right) + conc. sample\ 1\ \left(\mu\frac{g}{g}\right)}{3}\right)}{10{,}000\ (\mu\frac{g}{g})}\ x\ 100$$

Where conc. sample 1, 2 and 3 represent the concentration of additive (PBDE-209 or BPS) measured for the 1st, 2nd and 3rd replicate of sample, and 10,000 µg/g represent the total concentration of additive added to the polymer during manufacture.

Percentage of POPs leached in each solution

The percentage of PCBs leached in each solution was calculated as follow:

$$Percentage\ PCB\ leached\ (\%) = \frac{\left(\frac{conc. sample\ 1\ \left(n\frac{g}{g}\right) +\ conc. sample\ 2\ \left(n\frac{g}{g}\right) + conc. sample\ 1\ \left(\mu\frac{g}{g}\right)}{3}\right)}{\left(\frac{conc. hexane\ 1\ \left(n\frac{g}{g}\right) +\ conc. hexane\ 2\ \left(n\frac{g}{g}\right) + conc. hexane\ 3\ \left(\mu\frac{g}{g}\right)}{3}\right)}\ x\ 100$$

Where conc. sample 1, 2 and 3 represent the concentration of PCB (-28 or -138) measured for the 1st, 2nd and 3rd replicate of sample, and conc. hexane 1, 2 and 3 represent the concentration of PCB (-28 or -138) measured for the 1st, 2nd and 3rd replicate of hexane (different for each polymer).
