# Supplementary material for: Dynamics of leaching of POPs and additives from plastic in a Procellariiform gastric model: Diet- and polymer-dependent effects and implications for long-term exposure
Source: PLoS One. 2024 Mar 27;19(3):e0299860. doi: 10.1371/journal.pone.0299860 (PMC10971572; doi:10.1371/journal.pone.0299860)
Supplement: S1 Fig — (PDF) [file pone.0299860.s005.pdf]

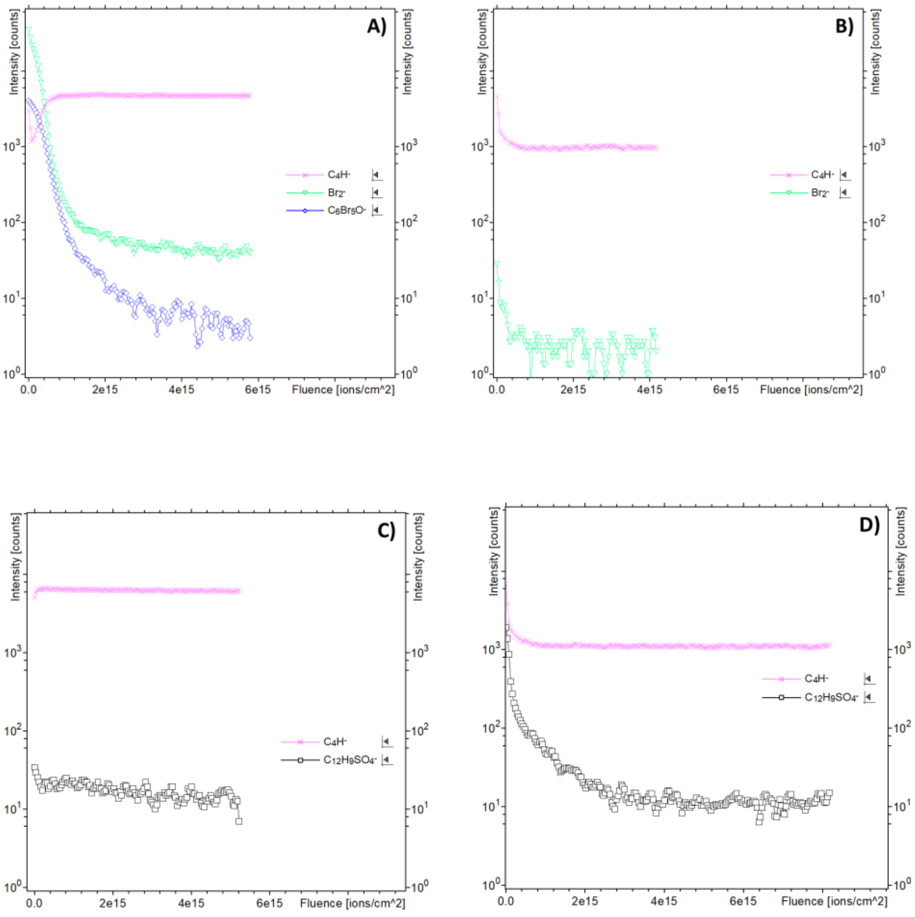

S1 Fig. Intensity of selected negatively-charged secondary ions recorded using ToF-SIMS upon depth-profiling on a) HDPE + PBDE209 ; b) PVC +PBDE209 ; c) HDPE + BPS and d) PVC + BPS. C4H- is mostly representing the polymers (HDPE and PVC), Br2- and C6Br5O- are related to PDBE209, C12H9SO4- is related to BPS. Note that C6Br5O- was not found in PVC+PBDE209. The conversion of fluence of the Argon GCIB used for surface erosion (see x-axis of the graphs) into a depth value is of the order of 58 nm/1015 ions/cm2 for HDPE and of the order of 37 nm/1015 ions/cm2 in the case of PVC. PBDE209 is enriched in a surface layer of about 180 nm in HDPE, while BPS is enriched in a surface layer of about 100 nm in PVC.
