# Supplementary material for: Dynamics of leaching of POPs and additives from plastic in a Procellariiform gastric model: Diet- and polymer-dependent effects and implications for long-term exposure
Source: PLoS One. 2024 Mar 27;19(3):e0299860. doi: 10.1371/journal.pone.0299860 (PMC10971572; doi:10.1371/journal.pone.0299860)
Supplement: S2 Fig — (PDF) [file pone.0299860.s006.pdf]

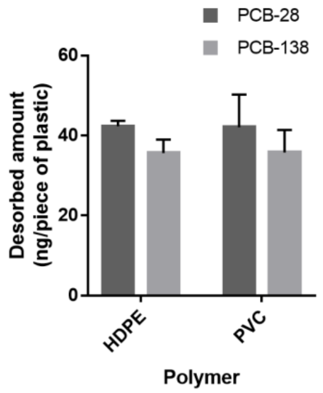

S2 Fig. Desorption of PCBs from HDPE and PVC in hexane after 20h. HDPE and PVC pieces containing 1% PBDE-209 and incubated for three weeks in salt water spiked with PCB-28 (dark grey) and -138 (light grey) (Fig 1) were leached to hexane for 20h at 38°C (800 rpm). Due to the difference in density between HDPE (0.96 g/cm³) and PVC (1.38 g/cm³), concentrations of PCBs released (ng per gram of plastic) were normalized and expressed as the amount (ng) released per one unit of a piece of plastic. Student's tests were applied to compare polymers. No differences were detected between the amount of PCB-28 (p-val = 0.96) and PCB-138 (p-val = 0.97) released from HDPE compared to PVC. The volume occupied by 42.2 ng of PCB-28 sorbed on a plastic particle represents 3.01 10^{-5} mm3 (density of PCB-28 = 1.4 mg/mm3) and the one occupied by 35.7 ng of PCB-138 sorbed on a plastic particle represents 2.23 10^{-5} mm3 (density of PCB-138 = 1.6 mg/mm3). Since one plastic particle represents a surface area of 70 mm^2 if the surface is considered to be smooth, it means that PCBs were most likely sorbed as a single layer onto plastic particles. Error bars represent standard deviation, n = 3.
