# Supplementary material for: Dynamics of leaching of POPs and additives from plastic in a Procellariiform gastric model: Diet- and polymer-dependent effects and implications for long-term exposure
Source: PLoS One. 2024 Mar 27;19(3):e0299860. doi: 10.1371/journal.pone.0299860 (PMC10971572; doi:10.1371/journal.pone.0299860)
Supplement: S3 Fig — (PDF) [file pone.0299860.s007.pdf]

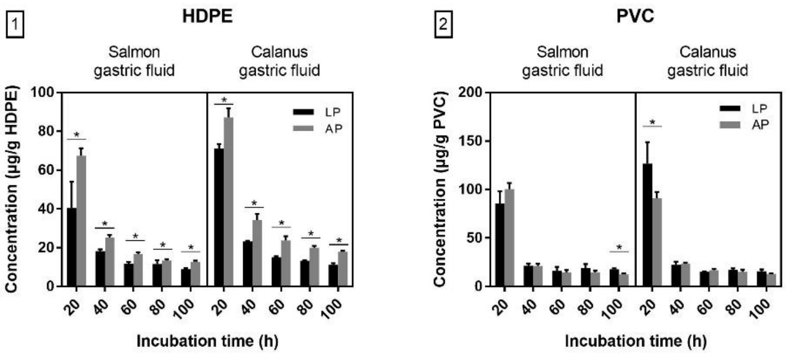

S3 Fig. Concentration of BPS released from HDPE (1) and PVC (2) in each phase of the salmon or calanus gastric fluid over time. Lipidic phases are shown in black ("LP") and aqueous phases are shown in grey ("AP"). Student's tests were applied to compare treatments and phases. Asterisk symbols (*) show significant differences between phases per hour (p-val < 0.05). Error bars represent standard deviation.
