# Supplementary material for: Dynamics of leaching of POPs and additives from plastic in a Procellariiform gastric model: Diet- and polymer-dependent effects and implications for long-term exposure
Source: PLoS One. 2024 Mar 27;19(3):e0299860. doi: 10.1371/journal.pone.0299860 (PMC10971572; doi:10.1371/journal.pone.0299860)
Supplement: S4 Fig — (PDF) [file pone.0299860.s008.pdf]

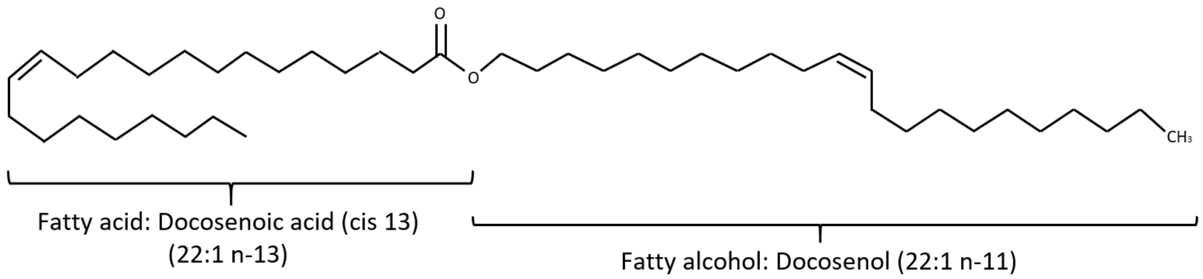

S4 Fig. Example of wax ester found in the oil of Calanus finmarchicus. This wax ester is composed of a long-chain polyunsaturated fatty acid docosenoic acid (cis 13) (C22:1 n-13) bound to a long chain fatty alcohol docosenol (22:1 n-11).
