# Supplementary material for: Dynamics of leaching of POPs and additives from plastic in a Procellariiform gastric model: Diet- and polymer-dependent effects and implications for long-term exposure
Source: PLoS One. 2024 Mar 27;19(3):e0299860. doi: 10.1371/journal.pone.0299860 (PMC10971572; doi:10.1371/journal.pone.0299860)
Supplement: S1 Table — (PDF) [file pone.0299860.s009.pdf]

S1 Table. Lipidic composition of the salmon and calanus oil.

Parameters	Concentrations	
	Salmon oil	Calanus oil
**Lipid class**		
Triglycerides	> 900 mg/g	3 mg/g (3)
Wax esters	NA	> 900 mg/g (2)
**Fatty acids**		
Total fatty acids	893 mg/g (1)	550 mg/g (2)
Saturated fatty acids	155 mg/g (1)	160 mg/g (2)
Myristic acid 14:0	26 mg/g (1)	64 mg/g (3)
Palmitoleic acid 16:0	94 mg/g (1)	45 mg/g (3)
Stearic acid 18:0	24 mg/g (1)	2 mg/g (3)
Monounsaturated fatty acids	425 mg/g (1)	150 mg/g (2)
Palmitoleic acid + iso 16:1 n-7	32 mg/g (1)	17 mg/g (3)
Oleic acid 18:1 n-9	326 mg/g (1)	16 mg/g (3)
Eicosenoic acid + iso 20:1 Gondoic acid 20:1 n-9	33 mg/g (1)	24 mg/g (3)
Docosenoic acid + iso 22:1 n-13 Cetoleic acid 22:1 n-11	4 mg/g (1)	43 mg/g (3)
Polyunsaturated fatty acids	313 mg/g	190 mg/g (3) - 240 mg/g (2)
n-3 fatty acids	152 mg/g (1)	> 180 mg/g (2, 3)
A-linolenic acid (ALA) 18:3 n-3	44 mg/g (1)	14 mg/g (3)
Stearidonic acid (SDA) 18:4 n-3	7 mg/g (1)	70 mg/g (2)
Eicosapentanoic acid (EPA) 20:5 n-3	35 mg/g (1)	65 mg/g (2)
Docosahexanoic acid (DHA) 22:6 n-3	43 mg/g (1)	50 mg/g (2)
n-6 fatty acids	155 mg/g (1)	> 30 mg/g(2)
Linoleic acid (LA) 18:2 n-6	140 mg/g	0.7 mg/g
**Fatty alcohols**		
Total fatty alcohols	NA	380 mg/g (2)
Of which are policosanols (sum 22:1 n-11, C20:1 n-9)	NA	> 210 mg/g (2)
Eicosenol 20:1 n-9	NA	188 mg/g (3)
Docosenol 22:1 n-11	NA	129 mg/g (3)

[1] Analysis performed in our laboratory.
[2] Calanus oil technical sheet (Zooca lipids©)
[3] Schots et al. (2020)
